# Involvement of Epithelial Na^+^ Channel in the Elevated Myogenic Response in Posterior Cerebral Arteries from Spontaneously Hypertensive Rats

**DOI:** 10.1038/srep45996

**Published:** 2017-04-06

**Authors:** Soo-Kyoung Choi, Soo-In Yeon, Youngin Kwon, Seonhee Byeon, Young-Ho Lee

**Affiliations:** 1 Department of Physiology, College of Medicine, Brain Korea 21 Plus Project for Medical Sciences, Yonsei University, Seoul, Korea

## Abstract

Hypertension is characterized by increased peripheral vascular resistance which is related with elevated myogenic response. Recent findings have indicated that epithelial sodium channel (ENaC) is involved in mechanotransduction of the myogenic response. The purpose of this study was to investigate the involvement of ENaC in the elevated myogenic response of posterior cerebral arteries (PCAs) from spontaneously hypertensive rats (SHRs). Sixteen to eighteen weeks old male wistar kyoto rats (WKYs) and SHRs were used in this study. We found that wall to lumen (W/L) ratio was increased in the PCAs from SHRs compared with WKYs at the resting state. Interestingly, amiloride significantly inhibited myogenic response in the PCAs from SHRs and WKYs, however, the magnitude of the blockade was greater in SHRs. The transfection of γENaC-siRNA significantly reduced the expression of γENaC protein and inhibited myogenic response in the PCAs from SHRs. Furthermore, these data were supported by the findings that serum/glucocorticoid-induced kinase (Sgk1) and neural precursor cell-expressed developmentally downregulated gene 4-2 (Nedd4-2) were increased in SHRs compared with WKYs. Our results suggest that γENaC may play an important role in the elevated myogenic response in PCAs from SHRs.

The myogenic response is an intrinsic response characterized by vasoconstriction in response to an increase of intraluminal pressure and vasodilation in response to a decrease of intraluminal pressure^1^. It is known that the myogenic response is inherent to smooth muscle and independent on the neural, metabolic, and hormonal influences^2^. The myogenic response has also been postulated to play an important role in establishing ambient vascular tone and auto-regulation of the blood flow in the resistance vasculature including coronary, cerebral, renal, and mesenteric arteries^3^,^4^,^5^,^6^. In these vessels, blood flow remains constant in spite of changes in perfusion pressure. Although it has been intensively investigated to clarify myogenic responses, the molecular mechanisms underlying the transduction of pressure into myogenic response remain unclear. Recently it has been suggested that mechano-sensitive ion channels in vascular smooth muscle cells may be the first step in the signal transduction of a stretch to vascular response^7^. The amiloride sensitive epithelial sodium channel (ENaC) is one of the candidate ion channels which are involved in cellular mechanotransduction^8^,^9^,^10^. The ENaC is a constitutively active channel which allows the flow of Na^+^ ions from the lumen into the epithelial cell, across the apical cell membrane^11^,^12^. Thus, the most well-known role of ENaC is related to Na^+^ reabsorption in many epithelia, such as the kidney, distal colon, secretory glands, and respiratory airways^12^. Because of their close evolutionary relationship to the C. elegans degenerins and their requirement for normal mechanosensory responses, ENaC proteins are considered to be components of mechanosensitive ion channel complexes in vertebrate tissue^13^. Furthermore, several studies reported that ENaC subunits are expressed in vascular smooth muscle cells and pharmacological blockade of ENaC reduced the vasoconstrictor response to increases of perfusion pressure^7^,^10^.

Hypertension is characterized by increased peripheral vascular resistance. Arterial wall thickening, increased vasoconstriction, and reduced vasodilation contribute to this exaggerated peripheral resistance^14^. Because arterial myogenic response is a component of vascular tone, this may contribute to the setting of total peripheral resistance and hence blood pressure regulation^14^. Several studies revealed that arterial myogenic response is increased in the arteries from hypertensive animals^15^,^16^. In the previous studies, we also have showed that the myogenic response is potentiated in the resistance arteries from hypertensive animals^17^.

In this study, we hypothesized that whether ENaC protein is involved in the potentiation of myogenic response in the posterior cerebral arteries (PCAs) from spontaneously hypertensive rats (SHRs). To test this hypothesis, we compared (1) the role of ENaC on myogenic response in PCAs from normotensive wistar kyoto rats (WKYs) and SHRs (2) expression level of ENaC protein in the PCAs from WKYs and SHRs and (3) effects of amiloride (specific ENaC inhibitor) at low micromolar dose and ENaC-siRNA on the pressure-induced myogenic response in isolated PCAs from WKYs and SHRs.

## Results

### Arterial structure-wall to lumen ratio

**Figure 1:**
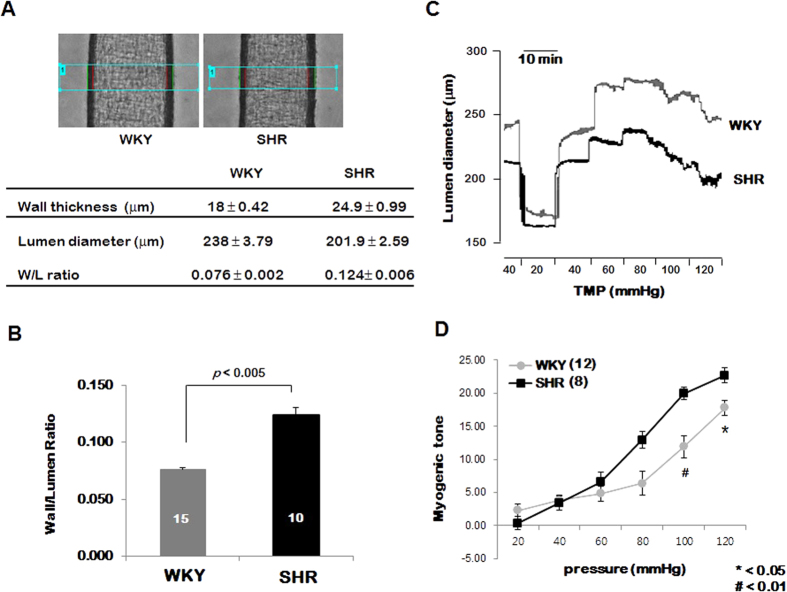
Comparison of the W/L ration in the PCAs from WKYs and SHRs. (**A**) Representative image showing the pressurized PCAs from WKYs and SHRs, and table showing the lumen diameter and W/L ratio. (**B**) Statistical data showing the W/L ratio. Data are expressed as means ± SEM (n = 15 in WKYs and n = 10 in SHRs). p < 0.05 for WKYs vs. SHRs. (**C**) Representative trace showing the myogenic response in the PCAs from WKYs and SHRs. (**D**) Mean data for the myogenic response in the PCAs from WKYs and SHRs. Data are expressed as means ± SEM (n = 12 in WKYs and n = 8 in SHRs) and are normalized to myogenic tone at a diameter of 40 mmHg. *p < 0.05 for WKYs and SHRs. #p < 0.01 for WKYs and SHRs.

The wall thickness and lumen diameter were measured while the arteries were pressurized at 40 mmHg of basal intraluminal pressure after the development of intrinsic tone at 36 °C. The Fig. 1A shows that the PCAs from SHRs showed significantly smaller lumen diameter (WKY vs. SHR: 238 ± 3.79 and 201.9 ± 2.59, respectively) and greater wall to lumen ratio than the PCAs from WKYs (Fig. 1B, WKY vs SHR: 0.076 ± 0.002 and 0.124 ± 0.006, respectively).

### Myogenic responses in the PCAs from SHRs and WKYs

The magnitudes of myogenic response were compared in PCAs from SHRs and WKYs. The inner diameter was measured while intraluminal pressure was increased in a stepwise manner from 20 mmHg to 120 mmHg in 20-mmHg increments. As shown in the Fig. 1C and D, myogenic response was elevated in the PCAs from SHRs compared with PCAs from WKYs.

### Effects of amiloride on myogenic response of PCAs from WKYs and SHRs

**Figure 2:**
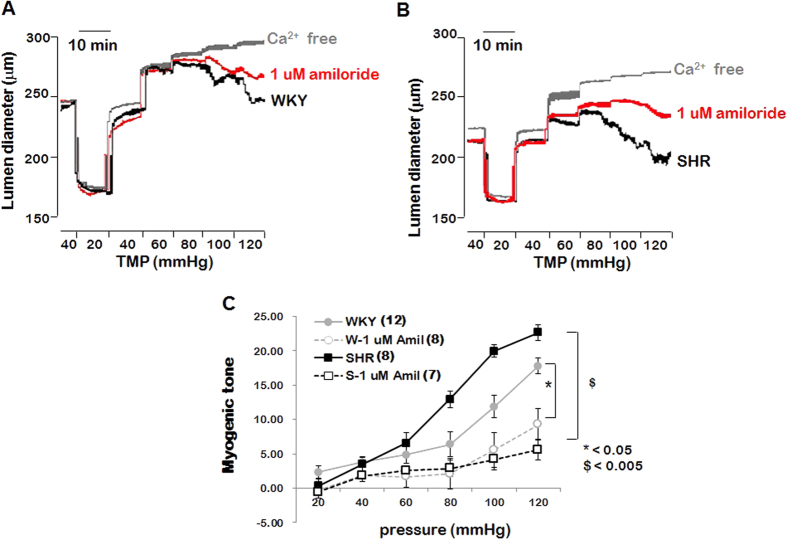
Comparison of amiloride effect on myogenic responses in the PCAs from SHRs and WKYs. (**A** and **B**) Representative recording showing the effect of 1 μmol/L amiloride on the myogenic response in the PCAs from WKYs and SHRs, respectively. (**C**) Mean data for the effect of amiloride. Data are expressed as means ± SEM and are normalized to myogenic tone at a diameter of 40 mmHg. *p < 0.05 for WKYs vs. WKYs with amiloride and ^$^p < 0.005 for SHRs vs. SHRs with amiloride. (n = 12 in WKYs, n = 8 in WKYs with amiloride, n = 8 in SHRs, and n = 7 in SHRs with amiloride).

**Figure 3:**
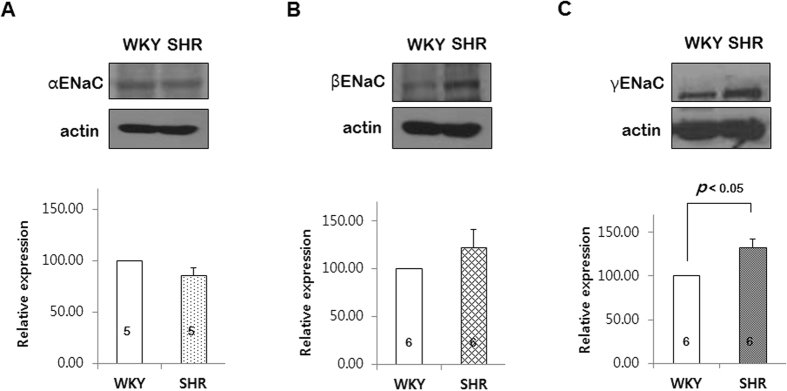
Comparison of ENaC protein subunits in the PCAs from SHRs and WKYs. The gels were run under the same experimental conditions while images of blots displayed in cropped format. (**A**–**C**) Western blots showing the expression of α-, β-, and γ-subunits of ENaC, respectively. Data are shown as means ± SEM. p < 0.05 for γ-subunits of ENaC. (n = 5 in αENaC, n = 6 in βENaC, and n = 6 in γENaC).

To test whether ENaC proteins are required for the myogenic response and compare the difference in the contribution to the myogenic response between WKYs and SHRs, we evaluated myogenic responses in isolated PCAs from WKYs and SHRs in the presence of amiloride (1 μmol/L, specific ENaC blocker). Figure 2A and B show the typical traces indicating effects of ENaC blockade on the myogenic responses in the PCAs from WKYs and SHRs, respectively. The amiloride significantly inhibited myogenic response in both groups (Fig. 3C). Interestingly, the magnitude of blockade was greater in the PCAs from SHRs compared with the PCAs from WKYs.

### ENaC protein subunits in the PCAs from SHRs and WKYs

Expression of ENaC subunits in the PCAs was measured using Western blots analysis. The anti α-, anti β-, and γ-subunits were detected in the PCAs from WKYs and SHRs (Fig. 3A,B and C, respectively). However, there was a significant difference between the PCAs from WKYs and SHRs only in the expression of γ-subunits (Fig. 3C).

### Effect of γ ENaC-siRNA on the protein and myogenic response in the PCAs from SHRs and WKYs

**Figure 4:**
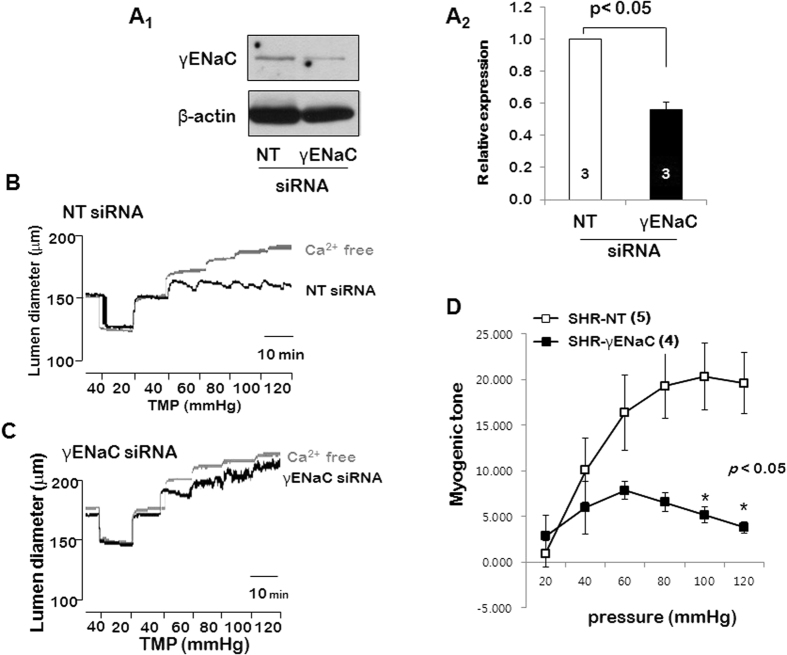
Effect of γENaC-siRNA on the γENaC protein expression and myogenic response in PCAs from SHRs. The gels were run under the same experimental conditions while images of blots displayed in cropped format. (**A**_**1**_) Western blots showing the effect of γENaC-siRNA on the expression of γ ENaC. β-Actin was used as a loading control. (**A**_**2**_) Mean data for the effect of γENaC-siRNA on the expression of γENaC protein. (**B**). Representative recording of myogenic response in PCAs transfected with NT-siRNA (**B**) or γENaC-siRNA (**C**). (**D**) Mean data for the effect of γENaC-siRNA on pressure-induced myogenic response in the PCAs from SHR. Data are shown as means ± SEM. *p < 0.05 for control (NT-siRNA) vs. transfected with γENaC-siRNA. (n = 5 in SHRs transfected with NT-siRNA and n = 4 in SHRs transfected with γENaC-siRNA).

To verify the contribution of γENaC on myogenic response in the PCAs from SHRs, we examined the myogenic response in siRNA-transfected PCAs from SHRs (Fig. 4). In western blots analysis, γENaC-siRNA significantly reduced endogenous protein level of γENaC after 3 days of transfection. In contrast, the protein level of γENaC did not change in the arteries transfected with NT-siRNA (Fig. 4A1 and A2). The myogenic response was measured in NT-siRNA- and γENaC-siRNA-transfected PCAs from SHRs. As shown in Fig. 4B and D, when the intraluminal pressure was raised from 20 to 120 mmHg in 20-mmHg increments, myogenic response was produced in NT-siRNA-transfected arteries, but transfection with γENaC-siRNA significantly inhibited the myogenic response (Fig. 4C and D).

### The expression of Sgk1 and phosphorylation of Nedd4-2 in the PCAs from SHRs and WKYs

**Figure 5:**
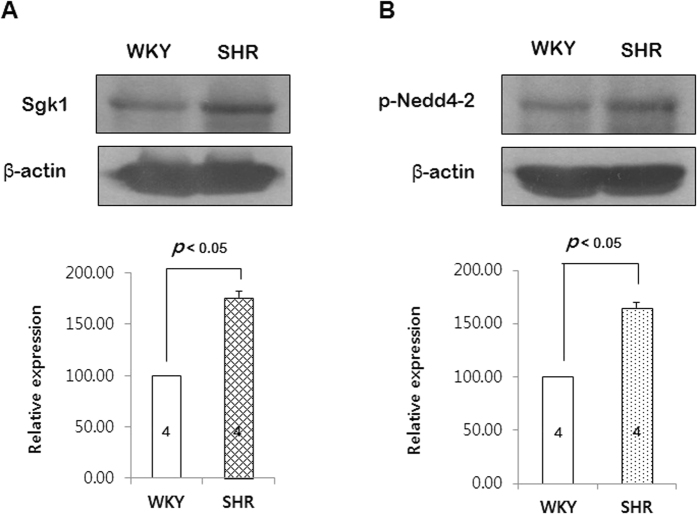
Expression of Sgk1 and phosphorylation of Nedd4-2 in the PCAs from SHRs and WKYs. The gels were run under the same experimental conditions while images of blots displayed in cropped format. (**A**) Western blots showing the expression level of Sgk1. (**B**) Western blots showing the phosphorylation level of Nedd4-2. p < 0.05 for WKYs vs. SHRs. Data are expressed 100% in WKYs. (n = 4 in Sgk1 and n = 6 in phospho-Nedd4-2).

To test whether the up-stream signaling molecules of ENaC change in the SHRs, we observed the expression of Sgk1 and phosphorylation of Nedd4-2 in the PCAs from WKYs and SHRs. As shown in the Fig. 5, expression level of Sgk1 was increased in the PCAs from SHR compared with those from WKY. Moreover, phosphorylation level of Nedd4-2 was also significantly increased in the PCAs from SHR compared with those from WKY.

## Discussion

This study demonstrates that myogenic response is potentiated in the PCAs from SHRs compared with those from WKYs, which is associated with increased expression of ENaC protein in SHRs. Interestingly, amiloride, the specific ENaC blocker, more significantly inhibited myogenic response in the PCAs from SHR compared with WKY. These data are in accordance with increased expression of γENaC in the PCAs from SHRs. To strengthen our data, the PCAs from SHRs were transfected with γENaC-siRNA. The transfection of γENaC-siRNA significantly reduced the expression of γENaC protein and inhibited myogenic response in the PCAs from SHRs. Furthermore, these data were supported by the findings that Sgk1 and phosphorylated Nedd4-2 which regulate ENaC expression were increased in SHRs compared with WKYs.

Hypertension is one of the major health problems worldwide. Hypertension is defined as elevations in blood pressure above 140 mmHg systolic or 90 mmHg diastolic and is an important risk factor for various diseases affecting brain, heart and kidneys^18^,^19^. Although much is known about the mechanisms regulating blood pressure, specific causes for hypertension in a minority of patients should be identified. The brain is a major target organ of the deleterious effects of hypertension and is responsible for a large portion of the mortality and morbidity^20^. It has been reported that there is a linear relationship between blood pressure and stroke mortality, and in patients with treated hypertension a 1 mmHg increase in systolic blood pressure increases stroke deaths by 2%^21^. Thus, finding new targets to alleviate hypertension could be significant therapeutic strategy.

The myogenic response plays an important role in autoregulation of blood flow of the resistance vasculature, especially in cerebral circulation because cerebral arteries are not particularly responded to the sympathetic nerves surrounding them^22^. Recently, several studies reported that the requirement of ENaC proteins for myogenic response^7^,^8^,^23^. In our previous studies, we also showed that the expression of ENaC protein and the effect of ENaC protein blockade with amiloride on myogenic response, and clarified that ENaC protein is critical for the mechanotransduction in the pressure-induced myogenic response^10^,^24^. However, the ENaC protein has been suggested as mechanotransducer, the involvement of ENaC in the elevated myogenic response in hypertension has not been investigated. Thus, this is the first study to investigate the involvement of ENaC in elevated myogenic response in the PCAs from SHRs.

We observed the wall/lumen ratio of PCAs in the WKY and SHRs, since W/L ratio is considered *in vivo* parameter of vascular structural remodeling which affects peripheral resistance. We found that W/L ratio was increased in the PCAs from SHRs compared WKYs, which indicates peripheral arterial resistance was increased in the SHRs (Fig. 1A and B). We also compared myogenic response in the PCAs from SHRs and WKYs. As shown in the Fig. 1C and D, myogenic response is significantly potentiated in the PCAs from SHR compared with those from WKYs. These data are in accordance with the studies revealed that in the resistance arteries, myogenic constriction is increased^14^,^15^. To define whether ENaC contributes to the elevated myogenic response in the SHRs, we compared the effect of amiloride on the myogenic response in the PCAs from WKYs and SHRs. As shown in the Fig. 2, amiloride significantly reduced myogenic response in both PCAs from WKYs and SHRs. Interestingly, the magnitude of blockade is greater in SHRs compared with WKYs. These data indicate that ENaC protein is involved in the myogenic response and contributes to the myogenic response more significantly in SHRs than WKYs. These data correlate with the previous studies showed blockade of myogenic response by amiloride^25^,^26^. In addition, this is the first study to compare the involvement of ENaC in the regulation of myogenic response in the PCAs from WKYs and SHRs.

We further investigated that which ENaC subunit is responsible for the elevated myogenic response in SHRs. The ENaC comprises three subunits, α, β, and γ^27^. In the previous report, we observed that β and γENaC showed relatively high expression level in resistance arteries of the rats^10^. Interestingly, in the present study, we found that only γENaC was significantly increased in the PCAs from SHRs (Fig. 3). Thus, we transfected the PCAs with γENaC- siRNA to reduce γENaC protein level. As shown in the Fig. 4, γENaC- siRNA reduced γENaC protein level by ~60% after 3 days of transfection. Moreover, transfection with γENaC-siRNA significantly inhibited pressure-induced myogenic response in the PCAs from SHR. Taken together, our results suggest that ENaC protein, especially γENaC plays an important role in the elevated myogenic response in SHRs. To strengthen our data, we investigated whether the modulators of ENaC degradation, Sgk1 and Nedd4-2, are also changed in the SHRs. It is known that the cell surface expression of ENaC is regulated by the ubiquitin protein ligase Nedd4-2. When Nedd4-2 is phosphorylated at Ser444, the interaction between Nedd4-2 and ENaC is reduced, which leads elevated cell surface expression of ENaC^28^. And Sgk1 is the upstream activator of Nedd4-2 which regulates the phosphorylation of Ser444^29^ In the present study, we found that expression of Sgk1was increased in SHRs compared with WKYs. In addition, phosphorylation of Nedd4-2 was also increased in SHRs (Fig. 5A and B). It has been well known that Sgk1 and Nedd4-2 regulate surface expression of ENaC in the renal tubular cell^30^,^31^,^32^, whereas it has not reported Sgk1 and Nedd4-2 regulation of ENaC in the vascular smooth muscle cell. In the present study, our data suggest that up-regulation of Sgk1 and Nedd4-2 may increase surface expression of ENaC in the PCAs from SHRs. To define exact mechanism, further studies are needed.

In summary, our results demonstrate that myogenic response is elevated in the PCAs from SHRs compared with those from WKYs, which is associated with increased expression of γENaC protein. Interestingly, γENaC-siRNA significantly reduced the expression of γENaC protein and inhibited myogenic response in the PCAs from SHRs. Furthermore, ENaC, Sgk1 and phosphorylated Nedd4-2 were also up-regulated in SHRs which is responsible for the increased surface expression of ENaC. Taken together, these results suggest that the γENaC may play an important role in the elevated myogenic response in PCAs from SHRs.

## Materials and Methods

All experiments were performed according to the Guide for the Care and Use of Laboratory Animals published by US National Institutes of Health (NIH publication No. 85-23, 2011) and were approved by the Ethics Committee and the Institutional Animal Care and Use Committee of Yonsei University, College of Medicine.

### Animals

Male WKYs and SHRs (16–18 weeks old; 300–350 g; Japan SLC, Inc., Hamamatsu, Shizuoka Prefecture, Japan) were used in this study. Animals were housed in a temperature- (25 ± 1 °C) and light-controlled (12:12-hour light:dark cycle) room with free access to water and normal rat chow (0.4% salt content). Rats were anesthetized with 5% isoflurane via a nose cone for euthanasia. The depth of anesthesia was monitored by pinching the toes, and no reaction was taken as a confirmation of proper anesthesia. The thoracotomy was used as a secondary method of euthanasia to ensure death.

### Tissue preparation

Rat brains were isolated and placed in ice-cold Krebs-Henseleit (K-H) solution composed of (in mmol/L): NaCl, 119; CaCl_2_, 2.5; NaHCO_3_, 25; MgSO_4_, 1.2; KH_2_PO_4_, 1.2; KCl, 4.6; and glucose, 11.1. The PCAs were excised from the brain, cleaned of connective tissue, and segmented into about 3–4 mm lengths. To eliminate the potential influence of endothelial factors on pressure-induced myogenic response, an air bolus was passed through the lumen to disrupt the endothelium. The PCA segments lacking a vasodilatory response to acetylcholine (1 μmol/L), but exhibiting efficient constriction to serotonin (10 μmol/L), were included in the studies.

### Measurement of myogenic response using an arteriograph

The PCAs were cannulated with glass micropipettes, and perfused with K-H solution bubbled with a 95% O_2_ + 5% CO_2_ gas mixture to maintain a pH of 7.4 at 36 °C in an arteriograph system (Living Systems Instrumentation, Burlington, VT, USA) as previously described^24^. The lumen diameter of PCAs was recorded using the SoftEdge Acquisition Subsystem (IonOptix, Milton, MA, USA). The PCAs were pressurized to 40 mmHg using pressure-servo control perfusion systems (Living Systems Instruments, St Albans, VT, USA) for a 40-minute equilibration period. After the equilibration period, the pressure was increased in a stepwise manner from 20 to 120 mmHg in 20-mmHg increments, and each pressure was maintained for 10 min to allow the vessel diameter to stabilize before measurements. At the end of the experiments, arteries were incubated with a 0-Ca^2+^ K-H solution containing the calcium chelating agent, EGTA (ethylene glycol tetraacetic acid, 1 mmol/L) and Ca^2+^ channel blocker nifedipine (5 μmol/L) to determine passive diameter. Responses to changes in intraluminal pressure were normalized as a percentage of the initial diameter at 40 mmHg to control for changes in the resting tone caused by the drugs. The following formula was used to calculate the percent myogenic response at each pressure step: percent myogenic tone = [(DpX/Dp40) −(DaX/Da40)] × 100, where DpX and Dp40 are the passive diameters at a given pressure and 40 mmHg in 0-Ca^2+^ K-H solution, and DaX and Da40 are the active diameters at a given pressure and 40 mmHg in normal K-H solution in the presence of extracellular Ca^2+^. *The* 1 μmol/L *of amiloride (highly specific dose for the ENaC*)^7^,^33^ *was used*.

### Western blot analysis

Freshly isolated PCAs from SHRs and WKYs were immediately frozen in liquid nitrogen and then homogenized in ice-cold lysis buffer. The expression of αENaC, βENaC, γENaC, Sgk1 (serum/glucocorticoid-induced kinase), and Nedd4-2 (neural precursor cell-expressed developmentally downregulated gene 4-2) proteins in PCAs was measured by western blot as previously described^34^. Briefly, for each preparation, vessels from 20 animals were pooled. Protein-matched samples (10 *μ*g protein/lane) were subjected to electrophoresis on 10% SDS-polyacrylamide gels and then transferred to nitrocellulose membranes. Membranes were incubated in 5% skim milk in PBS-Tween buffer for 2 h at room temperature and then incubated overnight at 4 °C in the presence of primary antibodies to αENaC, βENaC, γENaC, Sgk1, and Nedd4-2 antibody (1:200 dilution; Millipore, Darmstadt, Germany). Membranes were washed and then incubated with horseradish peroxidaseconjugated secondary antibody (1:1,000 dilution; Calbiochem, Darmstadt, Germany) for 1 h at room temperature. Immunoreactive bands were visualized by enhanced chemiluminescence (ECL; Amersham, Uppsala, Sweden). Developed films from ECL were scanned, and protein expressions were quantitated using Image J program (National Institute of Health, USA). Then, the membranes were stripped and re-probed using Western Blot Stripping Buffer for 10 min at 37 °C. (Thermo Scientific, Waltham, MA, USA) and incubated in 5% skim milk in PBS-Tween buffer for 2 h at room temperature and then incubated β-actin antibody (1:1000 dilution; Abcam, Cambridge, MA, USA) for verifying the equal loading between the samples.

### Small interfering RNA transfection of cerebral arteries

The PCAs were transfected with small interfering RNA (siRNA) as previously described^10^. Briefly, the arteries were removed in sterile conditions, cut into 3–4 mm segments and endothelium denuded. The segments from one artery were separated into two groups for transfection with NT- (non-targeting) siRNA or γENaC-siRNA (Bioneer Inc., Daejeon, Korea). The siRNA (10 nmol/L) was transfected with lipofectamine 2000 (Invitrogen, Carlsbad, CA, USA) according to the manufacturer’s instructions. After incubation for 5 h, PCA segments were immersed in Dulbecco’s modified Eagle’s culture medium supplemented with l-glutamine(1 mmol/L), penicillin (50 U/mL), and streptomycin (50 μg/mL) and maintained in an incubator (37 °C, air supplemented with 5% CO_2_) for 3 days. The duration of culture was determined on the basis of preliminary experiments to give a reproducible effect on artery contractile responses. Small interfering RNA duplexes were designed with RNAi designer software (Ambion, Austin, TX, USA) with accession number NM 017046 for γENaC. The NT-siRNA was purchased from Bioneer Inc. The sequences of siRNA duplexes were as follows: forward 5′-GGACCUGAUGCAUUGGUACdTdT-3′ and reverse 5′-GUACCAAUGCAUCAGGUCCdTdT-3′ for γENaC. The sequences of the NT-siRNA duplexes were forward 5′-CCUACGCCACCAAUUUCGUdTdT-3′ and reverse 5′-ACGAAAUUGGUGGCGUAGGdTdT-3′.

### Drugs

The following drugs were used: amiloride (Tocris Bioscience, Ellisville, MO, USA); acetylcholine chloride (Sigma-Aldrich, St Louis, USA); the general laboratory reagents (Sigma-Aldrich, St Louis, USA).

### Statistical analysis

Results are expressed as mean ± SEM. Comparisons between groups were performed with *t*-tests when the ANOVA test was statistically significant. Values of *P* < 0.05 were considered significant. Differences between specified groups were analyzed using the Student *t* test (2-tailed) for comparing 2 groups, with *P* < 0.05 considered statistically significant. For all experiments measuring diameter, the n-values mean number of vessels derived from each different animals. Accordingly, n-values also mean number of animals. For western blot analysis, all the PCAs from each group were pooled to obtain sufficient amount of protein, thus, the n-values mean number of experiments.

## Additional Information

**How to cite this article**: Choi, S.-K. *et al*. Involvement of Epithelial Na^+^ Channel in the Elevated Myogenic Response in Posterior Cerebral Arteries from Spontaneously Hypertensive Rats. *Sci. Rep.* **7**, 45996; doi: 10.1038/srep45996 (2017).

**Publisher's note:** Springer Nature remains neutral with regard to jurisdictional claims in published maps and institutional affiliations.
